# The Influence of Mass Media on the COVID-19 Vaccination Decision-making Process: Prospective Survey-Based Study

**DOI:** 10.2196/45417

**Published:** 2023-05-17

**Authors:** Cameron M Quon, Macey Walker, Lisa Graves

**Affiliations:** 1 Department of Family and Community Medicine Western Michigan University Homer Stryker MD School of Medicine Kalamazoo, MI United States

**Keywords:** accuracy, attitudes, behavior, communication, COVID-19, decision-making, dissemination, efficacy, employment, mass media, reliability, safety, survey study, usage, vaccination, vaccine hesitancy

## Abstract

**Background:**

Vaccine hesitancy during the COVID-19 pandemic was exacerbated by an infodemic of conflating accurate and inaccurate information with divergent political messages, leading to varying adherence to health-related behaviors. In addition to the media, people received information about COVID-19 and the vaccine from their physicians and closest networks of family and friends.

**Objective:**

This study explored individuals’ decision-making processes in receiving the COVID-19 vaccine, focusing on the influence of specific media outlets, political orientation, personal networks, and the physician-patient relationship. We also evaluated the effect of other demographic data like age and employment status.

**Methods:**

An internet survey was disseminated through the Western Michigan University Homer Stryker MD School of Medicine Facebook account. The survey included questions on media sources for COVID-19 information, political affiliation, presidential candidate choice, and multiple Likert-type agreement scale questions on conceptions of the vaccine. Each respondent was assigned a media source score, which represented the political leaning of their media consumption. This was calculated using a model based on data from the Pew Research Center that assigned an ideological profile to various news outlets.

**Results:**

The sample consisted of 1757 respondents, with 89.58% (1574/1757) of them choosing to take the COVID-19 vaccine. Those employed part-time and the unemployed were at 1.94 (95% CI 1.15-3.27) and 2.48 (95% CI 1.43-4.39) greater odds of choosing the vaccine than those employed full-time. For every 1-year increase in age, there was a 1.04 (95% CI 1.02-1.06) multiplicative increase in odds of choosing to receive the vaccine. For every 1-point increase in media source score toward more Liberal or Democrat, there was a 1.06 (95% CI 1.04-1.07) multiplicative increase in odds of choosing to take the COVID-19 vaccine. The Likert-type agreement scale showed statistically significant differences (*P*<.001) between respondents; those who chose the vaccine agreed more strongly on their belief in the safety and efficacy of vaccines, the influence of their personal beliefs, and the encouragement and positive experiences of family and friends. Most respondents rated their personal relationship with their physician to be good, but this factor did not correlate with differences in vaccine decision.

**Conclusions:**

Although multiple factors are involved, the role of mass media in shaping attitudes toward vaccines cannot be ignored, especially its ability to spread misinformation and foster division. Surprisingly, the effect of one’s personal physician may not weigh as heavily in one’s decision-making process, potentially indicating the need for physicians to alter their communication style, including involvement in social media. In the era of information overload, effective communication is critical in ensuring the dissemination of accurate and reliable information to optimize the vaccination decision-making process.

## Introduction

The World Health Organization (WHO) formally declared COVID-19 a pandemic on March 11, 2020 [1]. Since the beginning, the COVID-19 pandemic was met with an overabundance of information whether true, conflicting, false, or even harmful [1,2]. The WHO termed the volume of COVID-19 information an “infodemic,” which is described as a mixture of accurate and inaccurate information that makes it difficult for people to find trustworthy sources [3]. A preponderance of the COVID-19 rumors, stigma, and conspiracy theories circulating on the internet at the peak of the pandemic were shown to be false [4]. Despite this, there was a drop in health-related behaviors during the pandemic, including masking and social distancing [5]. The infodemic also contributed to psychological stressors with a spike in suicidal behavior and a rise in anxiety, fear, and depression particularly among women and young adults [6,7].

A global issue that became prominent during the COVID-19 pandemic is vaccine hesitancy, which the WHO defines as a “delay in acceptance or refusal of vaccination despite the availability of vaccination services,” which varies across time periods, places, and vaccines, and is influenced by complacency, convenience, and confidence [8]. During the COVID-19 pandemic, the infodemic challenged this confidence factor hindering the vaccine deployment process. In 2021, the US surgeon general predicted that the infodemic was the factor most likely to cause decreased vaccination rates for COVID-19 [9]. Exposure to information about COVID-19 on social media increases the likelihood of individuals believing misinformation [10]. A systematic review by Cascini et al [11] on social media attitudes toward the COVID-19 vaccination observed a higher vaccine acceptance rate in those using less social media.

Misinformation is often used to fill knowledge voids, leading to the distrust of science, pharmaceutical companies, and the government, as the media often employs the selective use of expert authority, straightforward explanations, the use of emotion, and “echo chambers,” which are information bubbles of tailored content that reinforce preexisting beliefs [12]. For example, political leaders and media outlets on the right and left have relayed divergent messages about the pandemic [13]. Members of the Republican and Democrat political parties demonstrate significant differences in COVID-19–related behaviors, such as social distancing and vaccination; a Pew Research Center study in 2020 showed greater vaccine hesitancy in populations with a more conservative political leaning with 69% (4969/7201) of adults with a Democrat leaning intending to get vaccinated compared to 50% (2572/5144) of those with a Republican leaning [13,14].

Vaccine hesitancy had been extensively explored well before the COVID-19 vaccine. The model produced by Dubé et al [15] shows that multiple historic, political, and sociocultural factors play a role in an individual’s decision-making process around vaccination decisions. These factors include knowledge and information, past experiences, the perceived importance of vaccination, risk perception and trust, subjective norms, and religious and moral convictions [15]. Whether a person refuses or accepts a vaccine is further modulated by trust in health professional recommendations, communication and media, and public health and vaccine policies [15].

The decision to receive the COVID-19 vaccine has been influenced by many of these same historical factors with those choosing to be vaccinated being White, male, older, Democrat, and having a higher income, education level, and increased trust in the government and health care professionals in addition to perceiving themselves to be at high risk of infection [10,16,17]. Those with more hesitancy tend to be a minority, pregnant or breastfeeding woman, younger, Republican or Independent, from a rural setting, have a lower income and education level, and hold past hesitancy about the safety and efficacy of vaccines [10,16,17].

If mass media overload lay on one side of the information spectrum, a constituent of the opposite side would be the individual patient-physician relationship, which can also strongly influence the perspective, behavior, and decisions of the patient. The importance of quality communication and a meaningful patient-physician relationship is seen in the literature as correlated with higher patient satisfaction, trust, and adherence to prescribed treatments [18]. Evidence shows that the quality of communication is important, especially as it relates to provider empathy levels, which are linked to better clinical outcomes (eg, in diabetic patients) [19]. For instance, in pediatric primary care practices, health care providers are consistently cited as key factors in a parent’s decision to vaccinate their children [20]. Recent data has shown that higher levels of trust in health care professionals and the government can counter the negative effect of misinformation and, therefore, increase the likelihood of COVID-19 vaccination in individuals [21].

In addition to the patient-physician relationship, an individual’s relationships with their networks are essential for multiple types of key health behavior changes, such as smoking cessation, weight loss, and HIV-risk mitigation [22]. When it comes to vaccination decisions, evidence indicates that individuals model the behaviors of their friends [23]. For example, a study by Rao et al [23] showed that students are more likely to get a flu shot if their friends did.

Although research has examined multiple factors leading to vaccine hesitancy, limited studies have investigated how specific media outlets have influenced this process. In this prospective survey-based study, we explore an individual’s decision-making process for the COVID-19 vaccine as it relates to the influence of specific media sources, with an additional investigation of political leaning, social networks, and the patient-physician relationship.

## Methods

### Selection and Description of Participants

In this prospective survey-based study, the public was solicited to fill out an internet survey on the REDCap system using a public internet link disseminated through the Western Michigan University Homer Stryker MD School of Medicine Facebook account with a post introducing the research project. The survey was open from August 1, 2021, to August 30, 2021.

Consent was requested at the beginning of the survey after details on the nature and objectives of the study were made available to the participants. Data were collected from all survey results received through the REDCap system. Participants were under no obligation to complete or submit the survey.

### Technical Information

The primary outcome was whether respondents chose to take the vaccine. Respondents were allocated into a group of participants that indicated they chose to take the vaccine if, on the survey, they selected any of the following: (1) they received the COVID-19 vaccine, (2) were scheduled to take the vaccine, (3) had taken the first dose and are waiting for the second dose, or (4) were fully vaccinated. Respondents were allocated into a group of participants that indicated they have not chosen to take the vaccine if, on the survey, they selected that they may take the vaccine or will not take the vaccine. Since the survey was at a single point in time, a portion of undecided participants may have later chosen to be vaccinated.

A media source use score was calculated using data from US adults from a survey conducted from October 29, 2019, to November 11, 2019, by the Pew Research Center, which solicited respondents on which media outlets they used and whether they identified as Democrat or Republican [24]. Our survey asked participants, “What news organizations do you use to get COVID information?” and listed the same media outlets from this study, which is freely available on the Pew Research Center website [24]. The question was phrased in this manner to appreciate that people may consume a media company’s information through a variety of sources, whether that is television, social media, radio, mobile apps, emails, newspapers, and more. Each media outlet was assigned a political leaning score based on the Pew Research Center’s data as a difference between the percentage of the audience who identified as Liberal or Democrat and the percentage identifying as Conservative or Republican. For example, if a respondent indicates using CNN (20), New York Times (37), and the Rush Limbaugh Show (80), the respondent will have a media source use score of (20+37+80)/3=−7.67. We note that the creation of this score has not been validated as a measure of the ideological profile, and some of the news sources indicated in the survey (Al Jazeera, Buzzfeed, CDC, etc) do not have an ideological profile measure and were scored as 0.

Additional covariates included political affiliation; presidential candidate choice; frequency of interaction with digital information; and demographic data (gender, education, employment status, and poverty screening). The survey also included a series of statements on a 7-point Likert-type agreement scale that assessed conception of COVID-19, vaccination, public health behavior, and the influence of their personal physician and those in their personal sphere of influence like friends and family (Table 1).

**Table 1:** Average values (SDs) for the 7-point Likert-type agreement scale items on vaccine perspectives stratified by COVID-19 vaccine choice.

Statement	Those choosing the COVID-19 vaccine (n=1574), mean (SD)	Those not choosing the COVID-19 vaccine (n=183), mean (SD)	*P* value^a^
Vaccines are safe.	6.38 (0.92)	4.60 (1.73)	<.001
The COVID-19 vaccine is safe.	6.20 (1.08)	2.45 (1.30)	<.001
The vaccine will protect me from COVID-19.	5.82 (1.24)	2.30 (1.54)	<.001
I am hesitant to get the vaccine because of my medical conditions.	1.29 (0.95)	2.52 (2.02)	<.001
I do not want to be vaccinated because of religious or moral reasons.	1.09 (0.58)	2.41 (2.07)	<.001
I am hesitant to seek medical care because I could get COVID-19 from the clinic or the hospital.	2.08 (1.50)	1.49 (1.11)	<.001
I have a good relationship with my doctor.	5.51 (1.60)	5.23 (1.82)	.05
I trust the expertise of local health experts such as my doctor and my hospital.	6.07 (1.21)	4.09 (1.73)	<.001
My family and friends encourage me to get the COVID-19 vaccine.	5.50 (1.73)	3.30 (1.85)	<.001
Most of my family and friends think people should get the COVID-19 vaccine.	5.72 (1.47)	3.34 (1.64)	<.001
Family and friends have had positive experiences with the COVID-19 vaccines.	5.99 (1.20)	3.38 (1.64)	<.001
Family and friends have had negative experiences with the COVID-19 vaccines.	2.19 (1.49)	4.27 (1.73)	<.001

^a^*P* values are computed using the 2-sample nonparametric Wilcoxon test for a difference in agreement between those choosing and those not choosing the COVID-19 vaccine with significance declared at a Bonferroni-corrected α of .0041.

### Statistics

Categorical data are reported as frequencies (percentages) and continuous data are reported as means (SDs). The prevalence of COVID-19 vaccination use was estimated using the total number of respondents indicating they have taken the vaccine out of the total number of survey respondents. A 95% CI was used to measure the precision of the estimate of the prevalence of COVID-19 vaccination.

To evaluate the association between media use source and vaccination, a logistic regression model was used—the media use source score was the independent variable, and vaccination choice was the dependent variable. The odds ratio was reported as a measure of the association between the media use source score and vaccination choice. The odds ratio indicates the multiplicative increase in the odds of choosing the vaccine as the media use score increases by 1 point toward a more Liberal or Democrat stance. The association between other demographic characteristics and vaccination choice was measured using univariate logistic regression. Significant univariate predictors were included in an adjusted model, and odds ratios and *P* values were reported. Significance was declared at α=.05. SAS v9.4 was used for data analysis. A full logistic regression model was built using variables found to be significant in the unadjusted models.

We also considered a subtractive regression model and found that the results were identical to the original outcome using backward stepwise selection.

### Ethics Approval

This study was reviewed and approved by the Institutional Review Board (IRB) of the Western Michigan University Homer Stryker MD School of Medicine, IRB number WMed-2021-0754, before data collection. The survey began with a statement on informed consent including that the study data are anonymous with no solicitation of identifying information. No compensation was provided for participation in or completion of the survey.

## Results

The sample consisted of 1757 respondents. Table 2 shows the distribution of the sample demographic characteristics by COVID-19 vaccination choice. The sample comprised 1574 participants who indicated that they chose to take the COVID-19 vaccine, with a prevalence rate of 89.58% (95% CI 88.16%-91.01%).

Table 1 shows the average values (SDs) for the 7-point Likert-type agreement scale (1=Strongly Disagree, 7=Strongly Agree) with items on vaccine perspectives, stratified by respondents who chose to take the COVID-19 vaccine. The left column includes the statements that were listed on the survey.

Respondents were asked about their experience with the COVID-19 vaccine. Of the participants, 32.49% (555/1708) indicated that their doctor had talked to them about the possible harms and benefits of the vaccine, 48.36% (828/1712) shared that their doctor had told them to get the vaccine, and 86.97% (1475/1696) reported that most of their family and friends had taken the vaccine or planned to do so.

Respondents were asked if they would consult a doctor for a variety of symptoms in the current state of the pandemic. Over half of the respondents indicated they would consult their doctor for new loss of taste or smell (1226/1757, 69.78%), shortness of breath (1307/1757, 74.39%), chest pain (1447/1757, 82.36%), weakness on one side of the body (1458/1757, 82.89%), loss of consciousness (1498/1757, 85.26%), or uncontrolled bleeding (1503/1757, 85.54%). Less than half of the respondents would consult their doctor for severe headache (846/1757, 48.15%), fever (733/1757, 41.72%), abdominal pain (541/1757, 30.79%), cough (471/1757, 26.81%), chills (428/1757, 24.36%), sore throat (345/1757, 19.64%), nausea or vomiting (305/1757, 17.36%), muscle or body aches (266/1757, 15.14%), generalized fatigue (245/1757, 13.94%), congestion (243/1757, 13.83%), or diarrhea (165/1757, 9.39%).

Respondents reported that they consulted their doctor in the last month (429/1757, 24.42%), 1-2 months ago (318/1757, 18.1%), 3-5 months ago (345/1757, 19.64%), 6 months to 1 year ago (439/1757, 24.99%), 2-5 years ago (143/1757, 8.14%), and 6-10 years ago (11/1757, 0.63%). A total of 4.1% (72/1757) of respondents shared that they do not have a personal doctor.

Respondents were asked to rate themselves on the political spectrum from liberal to conservative, with 1 being the most liberal and 7 being the most conservative; responses revealed the following trends: 1 (344/1756, 19.59%), 2 (358/1756, 20.39%), 3 (328/1756, 18.68%), 4 (291/1756, 16.57%), 5 (152/1756, 8.66%), 6 (89/1756, 5.07%), and 7 (89/1756, 5.07%). Additionally, 5.98% (105/1756) of respondents preferred not to answer, with one nonrespondent. Overall, most respondents identified as slightly left of center on the political spectrum, with a smaller percentage identifying as either more liberal or more conservative.

Respondents were asked to identify who they voted for in the 2020 US presidential election, with 69.78% (1212/1737) of respondents indicating that they voted for Joe Biden, 15.49% (269/1737) for Donald Trump, 2.82% (49/1737) for Joe Jorgensen, 1.44% (25/1737) for other candidates, and 0.12% (2/1737) for Howie Hawkins. Additionally, 4.15% (72/1737) of respondents reported that they did not vote in the election, and 6.22% (108/1737) indicated they preferred not to answer the question, with an additional 20 nonrespondents. Overall, most respondents indicated that they voted for Joe Biden, with a significant minority voting for Donald Trump and a small percentage voting for third-party candidates or indicating that they did not vote.

Respondents were asked to report how often they interact with digital information, such as through news, social media, television, and the internet. Of the participants, 0.34% (6/1755) reported never interacting with digital information, 0.4% (7/1755) interacting with digital information once a month, 1.99% (35/1755) once a week, 13.62% (239/1755) once a day, 72.19% (1267/1755) multiple times a day, and 11.45% (201/1755) multiple times each hour. Overall, most respondents indicated that they interact with digital information on a frequent basis, with a small minority reporting less frequent interactions.

**Table 2:** General demographic characteristics of the sample by COVID-19 vaccine choice.

Demographic characteristics			Those choosing the COVID-19 vaccine (n=1574), n (%)		Those not choosing the COVID-19 vaccine (n=183), n (%)		*P* value^a^	
**Education**								—^b^
	High school	23 (1.5)		11 (6.21)				
	Some college	229 (14.96)		40 (22.6)				
	Trade school	28 (1.83)		11 (6.21)				
	Bachelor’s degree	516 (33.7)		66 (37.29)				
	Some graduate school	112 (7.32)		12 (6.78)				
	Master’s degree	467 (30.5)		22 (12.43)				
	PhD or MD	80 (5.22)		5 (2.82)				
	Other education	76 (4.96)		10 (5.65)				
**Education by college degree**								<.001
	Less than bachelor’s degree or other education	356 (23.25)		72 (40.68)				
	Bachelor’s degree or Further	1175 (75.75)		105 (59.32)				
**Employment status**								.009
	Unemployed	261 (17)		15 (8.24)				
	Student	62 (4.04)		10 (5.49)				
	Part-time	231 (15.05)		17 (9.34)				
	Full-time	981 (63.91)		140 (76.92)				
**Was employment affected by COVID-19?**								.27
	No	810 (51.82)		98 (53.33)				
	Reduced pay	52 (3.33)		5 (2.73)				
	Reduced hours	168 (10.75)		16 (8.74)				
	Laid off	130 (8.32)		15 (8.2)				
	Increased hours	159 (10.17)		24 (13.11)				
	Increased pay	32 (2.05)		8 (4.37)				
	Respondent was unemployed	69 (4.41)		7 (3.83)				
	Other	143 (9.15)		10 (5.46)				
**Does money run out before you get to the end of the month?**								.02
	Yes	183 (11.92)		32 (17.78)				
	No	1352 (88.08)		148 (82.22)				
Age (years), median (IQR)			43 (34-56)		37 (28-47)		<.001	
**Gender**								.003
	Male	253 (16.1)		48 (26.23)				
	Female	1307 (83.2)		134 (73.22)				
	Nonbinary	11 (0.7)		1 (.55)				
**Race (missing n=22)**								.91
	White	1484 (95.31)		170 (95.51)				
	Of color	73 (4.69)		8 (4.49)				
**Do you know anyone who has had COVID-19? (Check all that apply)**								—
	No	39 (2.49)		6 (3.28)				
	Yourself	141 (8.99)		53 (28.96)				
	Family member	644 (41.07)		62 (33.88)				
	Close friend	277 (17.67)		23 (12.57)				
	Significant other	20 (1.28)		3 (1.64)				
	Acquaintance	196 (12.5)		16 (8.74)				
	Co-worker	171 (10.91)		11 (6.01)				
	Other	80 (5.1)		9 (4.92)				

^a^*P* values are computed using *t* test for age, and chi-square test for all survey items with mutually exclusive response options.

^b^Not available.

Table 3 presents the univariate and adjusted odds (95% CI) of the media source score and other respondent demographic characteristics based on COVID-19 vaccine choice.

For every 1-point increase in media source score toward a more Liberal or Democrat stance, there is an expected 1.06 (95% CI 1.04-1.07) multiplicative increase in the odds of choosing to take the COVID-19 vaccine. That is, those that tend to view media sources that are more Liberal or Democrat leaning in nature as measured by our formulation of ideological profile have a greater tendency of choosing to take the COVID-19 vaccine. The media score (*c*=0.77) is slightly less predictive than the political leaning scale (*c*=0.81), and when both are included in the model together, they remain significant, indicating that both the media score and the political leaning scale account for variation in vaccine choice independent of one another. The nonparametric Spearman correlation coefficient as a measure of the correlation between media score and the political leaning scale is ρ=−0.50.

Respondents employed part-time are at 1.94 (95% CI 1.15-3.27) greater likelihood of choosing to receive the COVID-19 vaccine than those employed full-time. Unemployed respondents are at 2.48 (95% CI 1.43-4.30) greater likelihood of choosing to receive the COVID-19 vaccine than those employed full-time. For every 1-year increase in age, there is an expected 1.04 (95% CI 1.02-1.06) multiplicative increase in odds of choosing to receive the COVID-19 vaccine.

**Table 3:** Unadjusted and adjusted association between respondent demographic characteristics with choosing to receive the COVID-19 vaccine.

		Unadjusted models			Adjusted model	
		Odds ratio (95% CI)	*P* value	Odds ratio (95% CI)		*P* value
Media score		1.05 (1.04-1.06)	<.001	1.06 (1.04-1.07)		<.001
Race of color (ref: White)		1.04 (0.49-2.20)	.91	—^a^		—
**Employment (ref: full-time)**						
	Part-time	1.94 (1.15-3.27)	.01	—		—
	Student	0.88 (0.44-1.77)	.73	—		—
	Unemployed	2.48 (1.43-4.30)	.001	—		—
Age		1.04 (1.02-1.06)	<.001	1.06 (1.04-1.08)		<.001

^a^Not available.

## Discussion

### Principal Findings

In this study, we examined factors associated with individuals’ decisions to receive the COVID-19 vaccine, with a specific focus on media sources and political leaning. We also explored the influence of the patient-physician relationship and close personal networks.

The relationship between demographic factors and vaccine uptake was not surprising. Regarding whether employment status was affected by COVID-19, no statistically significant difference was noted between those who chose to get the vaccine and those who did not. Yet, part-time employees and the unemployed were at greater odds of choosing the COVID-19 vaccine compared to full-time employees. Evidence on the relationship between employment and vaccine hesitancy is conflicting, with some showing unemployment being an impetus for vaccination as a prerequisite for job acceptance during the pandemic [17]. This contradicts research where the unemployed have reported lower influenza and COVID-19 vaccine acceptance compared to the employed, which could be confounded by education and income [25]. The US Bureau of Labor Statistics shows that as the years of education increase, income increases while unemployment rates decrease [26]. This is consistent with our survey results revealing statistically significant (*P*<.001) differences between those with a bachelor’s degree or higher compared to those without a bachelor’s degree. Social disadvantages, such as lower levels of income and education lead to poor access to accurate information, which has been cited as a driver for low confidence in the COVID-19 vaccine [27].

Our results on gender differences showed no significant difference in vaccination rates between male and female participants, differing from studies where women have reported a lower acceptance of the vaccine than men, especially for young women and pregnant or breastfeeding women, which was a distinguishing factor we did not explore [28]. Of note, most of our participants were at or approaching middle age and recipients of college education, which are 2 factors that predict greater rates of vaccine acceptance. This may have resulted in no significant gender difference.

Our results showing older respondents to be more likely to choose the vaccine compared to younger respondents is consistent with studies demonstrating that younger age groups tend to have a lower risk perception of COVID-19 [27].

Our results showed that those who consumed more Liberal or Democratic media and those who indicated a more Liberal or Democratic stance on the political spectrum were more likely to receive the vaccine. This is consistent with previous research and Centers for Disease Control and Prevention reports have shown that vaccination rates are higher in Democratic counties compared to Republican counties [29].

Unsurprisingly, our respondents heavily interacted with digital information, with a majority indicating its use multiple times a day (1267/1755, 72.19%), if not multiple times each hour (201/1755, 11.45%). As a measure of the influence that COVID-19 information had on our respondents, we asked them if they would consult a doctor upon the emergence of various symptoms including new loss of taste or smell, with 69.78% (1226/1757) indicating that they would. New change or loss in sense of smell is a hallmark symptom of the COVID-19 infection [30]. After a popular New York Times article linking loss of smell to the onset of a COVID-19 infection was published early in the pandemic, Tweets and Google searches regarding anosmia peaked for a week after the article was published at a frequency about 7 times higher than normal. The frequency of Tweets and Google searches related to anosmia was maintained at over 2.5 SDs from the typical frequency for weeks after the New York Times article publication [31]. These findings and our data suggest that media-informed knowledge of COVID-19 symptoms influences the public’s perceptions of the disease, which may affect their willingness to seek medical care.

The results of the Likert-type agreement scale showed that respondents who were more likely to take the COVID-19 vaccine had a stronger belief in vaccine safety and efficacy in general and, as such, in the COVID-19 vaccine (Table 1). They were also more hesitant to seek medical care due to the possibility of contracting COVID-19 at a medical facility, which suggests these respondents viewed the vaccine as a prophylactic measure. In addition, respondents were more likely to take the vaccine if it was in line with the beliefs, encouragement, and positive or negative experiences of their family and friends. This is consistent with evidence confirming a strong relationship between perceived vaccination social norms and the intention to get vaccinated, which declines as the referenced social group grows larger and more heterogeneous [32]. Respondents who chose not to get the vaccine were more strongly influenced by personal medical conditions and religious or moral reasons when compared to those who received the vaccine.

Most of the respondents (1531/1757, 87.14%) had consulted their doctor within the past year—a quarter of which were in the past month. All participants moderately agreed that they had a good relationship with their doctor (5.51 and 5.23, respectively, on the 7-point Likert-type agreement scale). However, no statistically significant difference was observed in vaccine choice between these 2 groups. Notably, only 48.36% (828/1712) of respondents indicated that their doctor encouraged them to get the vaccine and 32.49% (555/1708) talked about the possible harms and benefits. Regardless of whether the respondents spoke with their doctor on the issue, the influence physicians have on their patients’ decisions is challenged by the influence of social media, which the public may rely more heavily on for their sources of information, especially during public health crises when risk perception is elevated [33]. However, these 2 sources do not have to be mutually exclusive as physicians may also communicate with the public through media. However, a study published in 2021 found that less than 10% of tweets surrounding the COVID-19 vaccine came from medical professionals [34].

### Limitations

The respondent demographic may not have necessarily reflected the state or national vaccination status. Additionally, due to constraints set by our institution’s social media outreach policy, the survey was only posted on the institution’s Facebook page. During the survey period of August 2021, our survey group included 89.58% (1574/1757) of respondents who received at least one vaccine dose. This differed from the national vaccine status at the time, which was 61.8% (205,026,070/3,317,573,395) for the first dose and 52.4% (174,121,529/332,292,994) for series completion [35]. The vaccine status for Michigan was 55.2% (5,516,637/9,993,908) for the first dose and 50.5% (5,043,602/9,987,331) for series completion during this period [35].

The sample size was predominantly White race. This is important as racial and ethnic minorities in the United States are shown to have been more likely to report uncertainty or unwillingness to undergo vaccination [36]. Also, this study did not further distinguish between child-bearing-age and pregnant women as studies have shown pregnant or breastfeeding women to have greater vaccine hesitancy [37]. In the Likert-scale question on trusting in local health experts, both doctors and the hospital were grouped together to guard against survey fatigue; however, a split between the 2 categories may have better informed the patient-provider relationship.

Additionally, the political inclination of our respondents leaned more liberal, which was reinforced by more than two-thirds (1212/1737, 69.78%) of respondents indicating they voted for the Democratic candidate in the 2020 presidential election. Therefore, our sample size does not necessarily reflect the political preference of the country where 51.31% (81,268,924/158,383,403) voted for the Democratic candidate [38]. Meanwhile, the media score (*c*=0.77) was slightly less predictive than the political leaning scale (*c*=0.81). When both the media score and the political leaning scale are included in the model together, they remain significant, indicating that both measures account for variation in vaccine choice independent of one another.

In our measure of the frequency of the number of times respondents interact with digital information, it is important to note that this data may be difficult to generalize as some may do so for an extended period resulting in more interaction than those interacting multiple times an hour for brief moments. Additionally, some respondents may spend a greater proportion of time interacting with one media source versus another. Each participant’s media source use score is the average of their media sources’ political leaning score. We acknowledge that the media source use score is most accurate when participants interact with their media sources for an equal amount of time and is less accurate when participants favor some media sources more than others.

We recognize that by administering the survey through the medical school’s social media, we may be getting a unique group of respondents consisting of health-conscious individuals. However, we also know that this survey was open to the public during a time when social media engendered interaction with users who held both positive and negative perspectives about the vaccine [39].

Despite these limitations, conducting a survey such as this to gauge the social and media environment of the population served by the medical school fosters ongoing communication and outreach that aligns with the population’s needs. The insights gained from this study can be used to shape and improve how health care professionals and organizations approach patient care and prevention initiatives in the community. Conducting this study in local communities can yield insightful perspectives from the population, allowing physicians to tailor their communication approach accordingly.

### Conclusions

The COVID-19 pandemic has grown into a politically charged public health issue fueled by a media infodemic that affected various health behaviors, such as the decision to get vaccinated. When we examine our model that includes the variables of media score, political leaning, and the interaction between the two, we observe a significant interaction suggesting that media use impacts the effect political position has on vaccine choice.

Additionally, the results from our survey show that participants are influenced by their family and friends. In contrast, despite most respondents having a good relationship with their personal physician, there was no significant difference in vaccination decision likely indicating that physicians had less influence on their choice.

Future research could investigate specific messages and persuasive techniques that media outlets use to influence their audiences regarding health decisions, including algorithms on social media platforms. It could also help illuminate our findings as to why physicians are not as influential as the media, political leaning, and a person’s family and friends during a global pandemic. Additionally, further work could improve upon our ideological profile model by creating a more validated measure of a person’s political preference based on their choice of media companies. Future research can investigate how physicians might be better trained in social media competency and how physicians with a strong and positive public media presence operate. Physicians have the opportunity to interact with their patients not only in their practice but also through the media, which can improve their reach in countering false information. We urge other health institutions to undertake similar efforts to assess the information sources used by their local communities and adjust their communication approach accordingly.

The decision to receive the COVID-19 vaccine is a complex and multifactorial process that is influenced by various situational and socioeconomic factors. This is further complicated by the widespread availability of information about the vaccine, both accurate and inaccurate, which can make it difficult for individuals to make informed decisions about whether to take the vaccine. The mass media plays a critical role in disseminating this information as it can both inform and misinform the public about vaccine safety and efficacy. Effective communication is critical in ensuring that accurate and reliable information about the COVID-19 vaccine is available to the public to help individuals make informed decisions about whether to get vaccinated.

## Abbreviations

IRB: Institutional Review Board
WHO: World Health Organization

## Data Availability

The data sets generated and analyzed during this study are not publicly available due to IRB guidelines but are available from the corresponding author on reasonable request.
